# Bone Shape Feature Differences in Single and Recurrent Patellar Dislocations via Statistical Shape Modeling

**DOI:** 10.1002/jor.70243

**Published:** 2026-07-17

**Authors:** Mingrui Yang, John J. Elias, Mei Li, Jin Ma, Ceylan Colak, Carl S. Winalski, Lutul Farrow, Xiaojuan Li

**Affiliations:** ^1^ Program of Advanced Musculoskeletal Imaging (PAMI), Cleveland Clinic Cleveland Ohio USA; ^2^ Department of Biomedical Engineering Lerner Research Institute, Cleveland Clinic Cleveland Ohio USA; ^3^ Department of Health Sciences Cleveland Clinic Akron General Akron Ohio USA; ^4^ Department of Diagnostic Radiology Mayo Clinic Rochester Minnesota USA; ^5^ Department of Diagnostic Radiology Cleveland Clinic Cleveland Ohio USA; ^6^ Department of Orthopaedic Surgery Cleveland Clinic Cleveland Ohio USA

**Keywords:** magnetic resonance imaging, osteoarthritis, patellar dislocation, patellofemoral, statistical shape modeling

## Abstract

This study aims to develop MRI‐based 3D statistical shape models for patellar dislocation patients and investigate bone shape features that distinguish between single and recurrent dislocation populations, as well as control and patellar dislocation populations. MRIs from 16 single dislocation patients, 17 recurrent dislocation patients, and 20 control subjects were used to build statistical shape models for femur and patella bones. Bone shape features were extracted and tested for statistical significance (*p* < 0.05) for distinguishing the single dislocation group from the recurrent dislocation groups, and the control group from the dislocation group. Statistically significant shape features distinguishing the three groups were identified for both bones. A shallower trochlear groove and smaller patellar ridge told the dislocation groups apart from the control group. A prolonged lateral condyle in femur and a laterally shifted ridge and hood shaped lateral facet in patella distinguished the recurrent dislocation group from the single dislocation group. The study's findings from 3D statistical shape modeling of the femur and patella reveal distinct anatomical differences across control, single dislocation, and recurrent patellar dislocation groups. This advanced understanding aids in identifying individuals at higher risk for initial or recurring patellar dislocations, enabling clinicians to tailor treatment and management strategies more effectively to prevent further dislocations and mitigate potential patellofemoral joint damage.

## 1 Introduction

Patellar dislocation is a traumatic injury caused by direct impact to or severe twisting of a knee that may cause significant functional impairment to a young, active patient population. The incidence of acute patellar dislocation in the highly active young population of age 10–17 has been estimated to be 4 times more than in the general population [1]. Roughly over 60% of these dislocations occur during active exercise or sports [1]. Females face an elevated risk of patellar dislocation, experiencing an approximately 33% higher incidence compared to males [1]. This poses a significant challenge for young athletes due to the substantial recurrence rates, ranging from 15% to 44% [2, 3]. Notably, individuals with a history of dislocation are seven times more likely to encounter further episodes of instability [1, 4, 5]. Moreover, patellar dislocation frequently leads to patellofemoral osteoarthritis (OA). A recurrent dislocation further increases the risk of developing OA by a factor of 4.5 as compared to a single dislocation [6]. The likelihood and grade of cartilage lesions increase with the time following the initial injury [7].

Both conservative management and surgical procedures have been used to treat patellar dislocation patients [8]. Conservative management options include dynamic strengthening via physical therapy and bracing, which have been used for treating single dislocation patients. For recurrent dislocation patients where conservative treatment options failed, surgical interventions, such as medial patellofemoral ligament (MPFL) reconstruction, lateral retinaculum lengthening, and tibial tubercle osteotomy, have been shown to provide some satisfactory patient outcomes and post‐operative stability [8, 9, 10, 11, 12, 13]. Improved understanding of anatomical features of the patellofemoral joint that contribute to dislocations has been the driving force for tailored treatment plans for patellar dislocation patients [14]. Although anatomic risk factors have been identified for multiple dislocations, including a shallow trochlear groove, a high riding patella, and a lateral position of the tibial tuberosity [15, 16, 17, 18, 19], the specific features that indicate highest risk of additional dislocations following the first injury are not yet clear. Improved understanding of anatomic characteristics that compromise patellar stability is needed to optimize early intervention to minimize additional dislocations and OA development, without performing unnecessary surgery.

Statistical shape modeling (SSM) is a powerful method in modeling object shape variations that dates back to the 1990s [20]. It has been adapted to bone structures in the knee joint for various studies, such as kinematic tracking and bony differences in osteoarthritic knees based on radiograph and CT images [21, 22]. With the increased availability of high‐quality medical imaging data in the last decade, MRI has become increasingly important for bone SSM and joint kinematic analyses, especially for the knee joint. Knee MRI‐based SSM has shown the capability of extracting data‐driven shape features to provide the opportunity to consider subject‐specific 3D bone anatomy when diagnosing pathologies, designing implants, making surgical decisions, and predicting patient‐specific outcomes [23, 24, 25, 26, 27, 28, 29].

This study aims to develop MRI‐based 3D SSM for patellar dislocation patients and investigate data‐driven femur and patellar bone shape features that distinguish between control and patellar dislocation populations. More importantly, data‐driven femur and patellar bone shape feature differences between the single and recurrent dislocation patients are also explored.

## 2 Methods

This study is a prospective observational cohort study with Level of Evidence III. The study was approved by the Cleveland Clinic Institutional Review Board (IRB # 18‐1388) and all subjects (and their guardians for minors) signed a consent or assent form prior to participating.

### 2.1 Subjects

Patients were recruited based on treatment for a complete traumatic dislocation of the patella from the trochlear groove for one knee. Exclusion criteria were: (1) age less than 13 years, (2) prior surgery for either knee, and (3) more than 6 months since the most recent dislocation. Thirty‐five injured subjects (24 females) with age between 13 and 37 years old (20 ± 6.8), and BMI between 16.2 and 45.5 kg/m^2^ (25.3 ± 6.3), who had at least one traumatic patellar dislocation episode within 6 months were prospectively recruited from January, 2019 to June, 2022. All dislocations were confirmed by identification of a bone bruise on the medial patella and lateral femoral condyle on MRI. Single dislocation subjects who experienced additional dislocations during study period were re‐classified. Seventeen of these subjects (13 females) had self‐identified, MRI‐confirmed multiple dislocations. An additional 20 subjects (10 females) between the ages of 13 and 33 years (19.5 ± 6.2), and BMI between 19.4 and 38.4 kg/m^2^ (24.1 ± 4.6) with no history of prior surgeries, or injuries for either knee were recruited to participate in the control group.

### 2.2 MRI

MRI scans were collected on a 3 T scanner (Prisma, Siemens Healthineers) using a 1Tx/15Rx knee coil (Quality Electodynamics). At least one knee (injured side) for the patients, and one knee for the controls was scanned. The MRI protocol included clinical sequences with turbo spin echo (TSE) images, and a 3D sagittal non‐fat saturated Sampling Perfection with Application optimized Contrasts using different flip angle Evolution (SPACE) sequence for bone segmentation and shape analysis. The major parameters of SPACE included field of view of 152 mm × 160 mm, number of slices of 176, isotropic 3D resolution of 0.5 mm, repetition time of 1000 ms, and echo time of 28 ms with variable flip angles. The average scan time of SPACE was 4 min and 55 s.

### 2.3 Bone Shape Modeling

**Figure 1:**
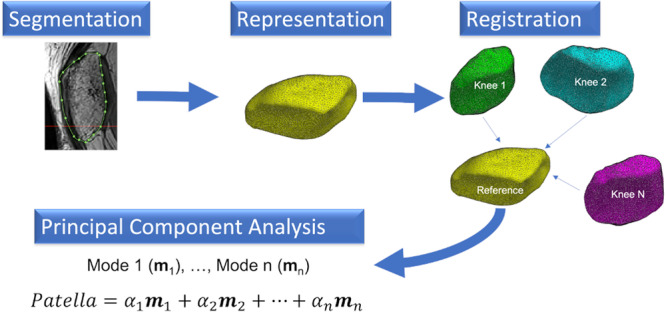
Bone shape modeling pipeline diagram. The bone statistical shape modeling pipeline can be divided into bone segmentation, bone shape representation selection, bone shape registration, and bone shape model construction using, e.g., principal component analysis.

The bone shape modeling pipeline consisted of bone segmentation, mesh generation, bone surface registration, and bone shape model construction using principal component analysis (Figure 1). Femur and patella were first automatically segmented using an in‐house developed deep learning‐based segmentation tool [30] transferred to the non‐fat saturated SPACE MR images. The bone segmentations were further quality controlled by a trained reader who reviewed the segmentation for all cases and performed manual corrections as needed. Binary segmentations were converted to triangular surface meshes. Each mesh was smoothed using curvature‐flow Laplacian smoothing with duplicate vertices, duplicate triangles, and degenerate faces removed. No downsampling was applied to the high‐density meshes. Moving meshes with fewer vertices than the selected reference mesh were repeatedly subdivided using a Shirman opposite‐edge 4‐split until their vertex counts matched the reference mesh.

Registration was performed separately for the femur and patella. For each bone, all control meshes were used as candidate references, and the control mesh with the smallest average registration error was selected as the reference for subsequent registration and analysis. Thus, the femur and patella reference meshes were selected independently and could come from different control knees. For each candidate reference, the remaining control meshes were registered using FOCUSR [31] to estimate surface correspondence in the Laplace‐Beltrami eigen basis, followed by coherent point drift alignment in spectral space. The selected correspondence points were then registered to the reference point cloud using Procrustes registration with isotropic scaling [32, 33], followed by the rigid iterative closest point (ICP) registration [34] using point‐to‐point minimization.

Separate statistical shape models were constructed for the distal femur and patella. For each bone, the registered subject‐specific point clouds from the control, single‐dislocation, and recurrent‐dislocation groups were combined, the mean shape was calculated, and principal component analysis was applied to identify orthogonal modes of variation. The modes of variation (eigenvectors) were ordered by the corresponding variances (eigenvalues).

Each bone from all groups was represented as a linear combination of these modes. The control, single‐dislocation, and recurrent‐dislocation groups were compared pairwise across retained femur and patella modes that together accounted for at least 95% of shape variance. Group differences were tested using rank‐based regression adjusted for age, sex, and BMI. Rank‐based regression was chosen because of the modest cohort size and because principal‐component scores were not assumed to be normally distributed. Because this was an exploratory study intended to identify candidate shape features for future validation, statistical significance was evaluated using nominal *p* < 0.05 without correction for multiple comparisons.

## 3 Results

A total of 27,204 surface points for each femur were chosen as the representation points for shape registration. Similarly, the representation points cloud for each patella surface has a size of 6182 due to its relatively smaller size. All femur and patella bones were registered successfully to the reference bone surfaces, respectively.

**Figure 2:**
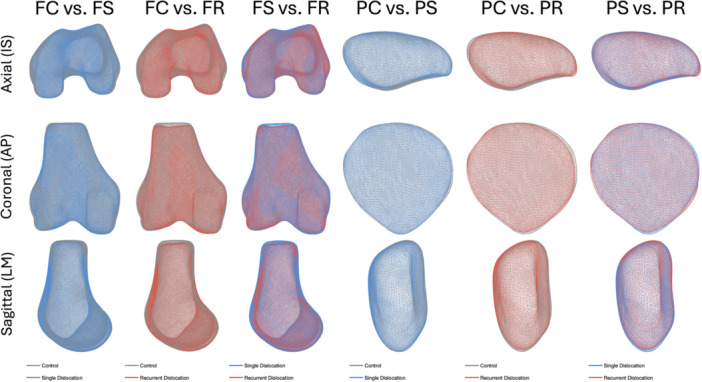
Pairwise comparison of mean surface meshes for the femur (left) and patella (right) among the control (FC/PC, gray), single‐dislocation (FS/PS, blue), and recurrent‐dislocation (FR/PR, red) groups. For each bone, the three rows show the axial view, with the inferior‐to‐superior direction into the plane (IS); the coronal view, with the anterior‐to‐posterior direction into the plane (AP); and the sagittal view, with the lateral‐to‐medial direction into the plane (LM). The three columns show pairwise comparisons of the group mean shapes. All panels share the same triangulation and are displayed at a matched anatomical scale. For the femur, the dislocation groups show narrower femoral condyles and shallower trochlear grooves compared with the control group. For the patella, the dislocation groups show a more convex medial facet compared with the control group, and the recurrent‐dislocation group shows a more concave lateral surface compared with the other two groups.

The mean surface meshes for femur and patella are shown in Figure 2, where the mean shape of the control subjects is shown in gray, the mean shape of the single‐dislocation patients is shown in blue, and the mean shape of the recurrent‐dislocation patients is shown in red. Each bone is displayed in axial, coronal, and sagittal views with matched anatomical scale. For femur, the single and recurrent dislocation groups had smaller condyle widths but vertically stretched medial and lateral condyles, and shallower trochlear grooves compared to the control group. For patella, the dislocation groups showed a more convex medial facet compared to the control group. The lateral facet of the recurrent dislocation group exhibited a more concave surface resulting in a hook shape compared to the control and single dislocation groups.

PCA was applied to the whole population for femur and patella separately, resulting in more than 50 modes in total, respectively. The first 20 modes covered at least 95% of the mode variations for both femur and patella shape models. Therefore, we expressed each individual bone as a linear combination of these 20 modes and examine the distribution of the coefficients of each mode for femur and patella respectively.

As shown in Table 1, the mean coefficients for modes 4, 5, 8 and 19 of the femur bone had statistically significant (*p* < 0.05) differences among the study groups; while the mean coefficients for modes 1, 3, 8, 16 and 17 of the patella bone had statistically significant (*p* < 0.05) differences among the study groups.

**Table 1:** Pairwise comparison of the mean (±standard deviation) principal component scores for femur and patella modes of SSM among the control, single dislocation, and recurrent dislocation groups. A significance level of *p* < 0.05 was indicated by *; a significant level of *p* < 0.01 was indicated by **. Note that a “−” indicated that the group was excluded from that paired comparison.

Femur	Control	Single Dislocation	Recurrent Dislocation	*p*‐value
Mode 4	93.06 ± 127.37	231.80 ± 137.10	—	0.002 (**)
	93.06 ± 127.37	—	182.57 ± 88.14	0.012 (*)
Mode 5	−872.54 ± 105.59	−786.04 ± 123.83	—	0.011 (*)
	−872.54 ± 105.59	—	−822.52 ± 103.98	0.025 (*)
Mode 18	−444.27 ± 38.94	−420.00 ± 23.27	—	0.028 (*)
		−420.00 ± 23.27	−437.23 ± 48.64	0.049 (*)
Mode 19	—	−391.25 ± 31.33	−358.17 ± 30.06	0.014 (*)

**Patella**	**Control**	**Single Dislocation**	**Recurrent Dislocation**	**p‐value**
Mode 1	69.23 ± 74.90	11.30 ± 74.79	—	0.048 (*)
	69.23 ± 74.90	—	−22 ± 91.88	0.012 (*)
Mode 3	46.42 ± 35.55	—	2.13 ± 51.14	0.022 (*)
	—	53.96 ± 53.57	2.13 ± 51.14	0.014 (*)
Mode 8	66.01 ± 18.21	59.38 ± 26.95	—	0.047 (*)
Mode 16	—	−18.03 ± 6.59	−9.89 ± 10.53	0.013 (*)
Mode 17	14.43 ± 11.34	21.81 ± 8.84	—	0.048 (*)

**Figure 3:**
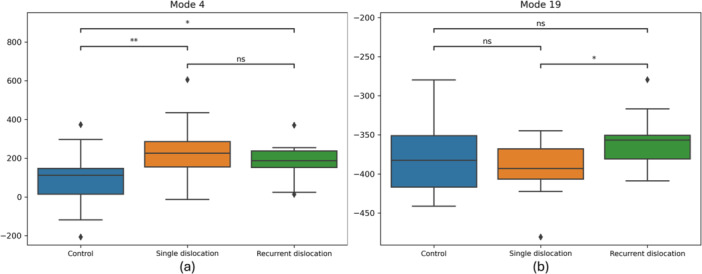
Boxplots of mode 4 and 19 for femur. (a) Boxplots comparing femur mode 4 coefficients of different groups. (b) Boxplots comparing femur mode 19 coefficients of different groups. Mode 4 was significant in differentiating the control group from the dislocation groups. Mode 19 was significant in differentiating the single dislocation group from the recurrent dislocation group.

**Figure 4:**
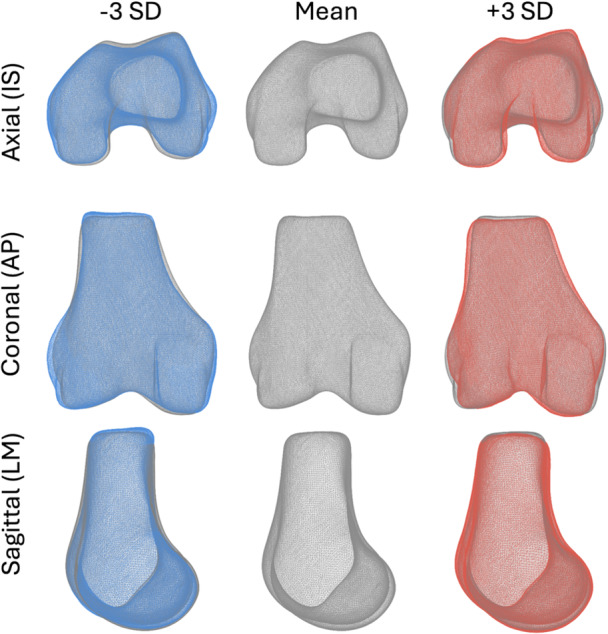
Femur mode 4 surface deformation rendered at −3 SD (blue, left), the population mean (gray, center), and +3 SD (red, right). Rows show axial (IS, top), coronal (AP, middle), and sagittal (LM, bottom) views. Each deformed surface is drawn on top of a faint wireframe of the population mean shape, so the displacement is visible against a shared reference. The +3 SD direction illustrates a more prominent lateral anterior femoral condyle and a shallower trochlear groove.

In particular, mode 4 of femur was a significant mode that differentiated the control group from the single and recurrent dislocation groups, as shown in the boxplot in Figure 3a. Specifically, the single and recurrent dislocation groups had significantly larger (*p* < 0.01) mode 4 coefficients compared to the control group. Figure 4 shows the femur mode 4 deformation as −3 SD, mean, and +3 SD surface meshes with the mean shape overlaid. The +3 SD direction illustrates a more prominent lateral anterior femoral condyle and a shallower trochlear groove surface, consistent with the larger mode 4 coefficients in the dislocation groups.

**Figure 5:**
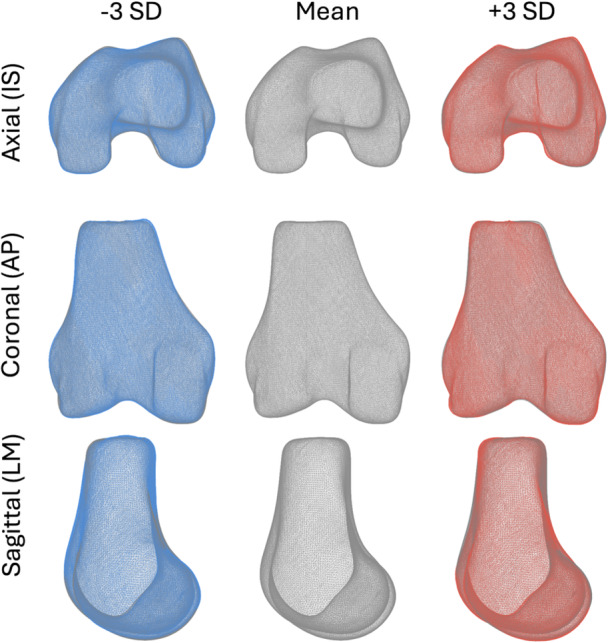
Femur mode 19 surface deformation rendered at −3 SD (blue, left), the population mean (gray, center), and +3 SD (red, right). Rows show axial (IS, top), coronal (AP, middle), and sagittal (LM, bottom) views. Each deformed surface is drawn on top of a faint wireframe of the population mean shape so the displacement is visible against a shared reference. The +3 SD direction illustrates a prolonged lateral femoral condyle head.

Mode 19 was the mode that differentiated the single dislocation group from the recurrent one as shown in the boxplot in Figure 3b. Specifically, the recurrent dislocation group had significantly larger (*p* < 0.05) mode 19 coefficients compared to the single dislocation group. Figure 5 shows the femur mode 19 deformation as −3 SD, mean, and +3 SD surface meshes with the mean shape overlaid. The +3 SD direction illustrates a prolonged lateral femoral condyle head in the anterior‐posterior direction. Therefore, the recurrent dislocation group had significantly larger lateral femoral condyle depth along the anterior‐posterior direction compared to the single dislocation group.

**Figure 6:**
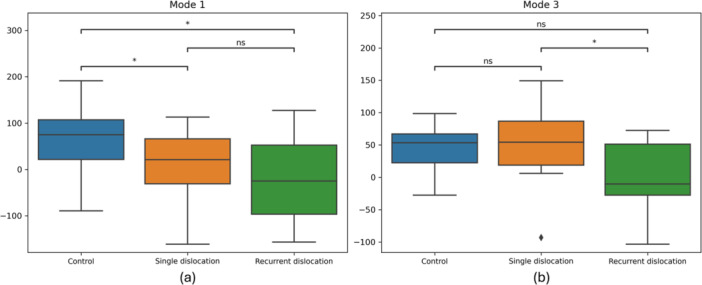
Boxplots of mode 1 and 3 for patella. (a) Boxplots comparing patella mode 1 coefficients of different groups. (b) Boxplots comparing patella mode 3 coefficients of different groups. Mode 1 was significant in differentiating the control group from the dislocation groups. Mode 3 was significant in differentiating the recurrent dislocation group from the control and single dislocation groups.

**Figure 7:**
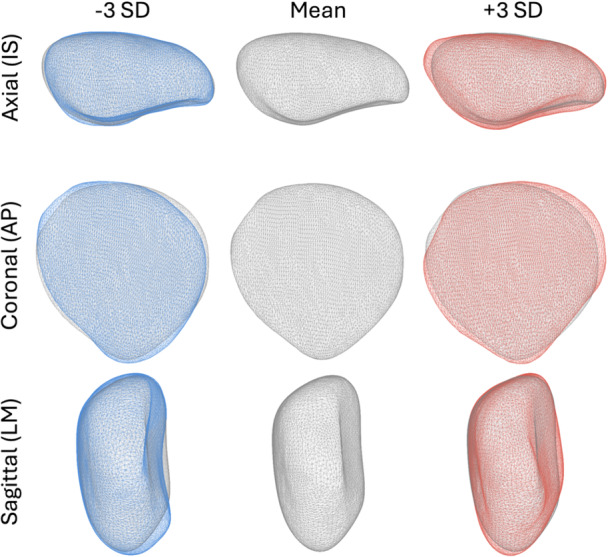
Patella mode 1 surface deformation rendered at −3 SD (blue, left), the population mean (gray, center), and +3 SD (red, right). Rows show axial (IS, top), coronal (AP, middle), and sagittal (LM, bottom) views. Each deformed surface is drawn on top of a faint wireframe of the population mean shape so the displacement is visible against a shared reference. The −3 SD direction illustrates a shorter and more convex medial facet, a more concave lateral facet and a thinner patellar ridge.

For patella, mode 1 was a statistically significant (*p* < 0.05) mode that separated the control group from the single and recurrent dislocation groups, as shown in Figure 6a. Specifically, the control group had significantly larger mode 1 coefficients compared to the single and recurrent dislocation groups. Figure 7 shows the patella mode 1 deformation as −3 SD, mean, and +3 SD surface meshes with the mean shape overlaid. The −3 SD direction illustrates a more concave lateral facet and a thinner patellar ridge, consistent with the lower mode 1 coefficients in the dislocation groups.

**Figure 8:**
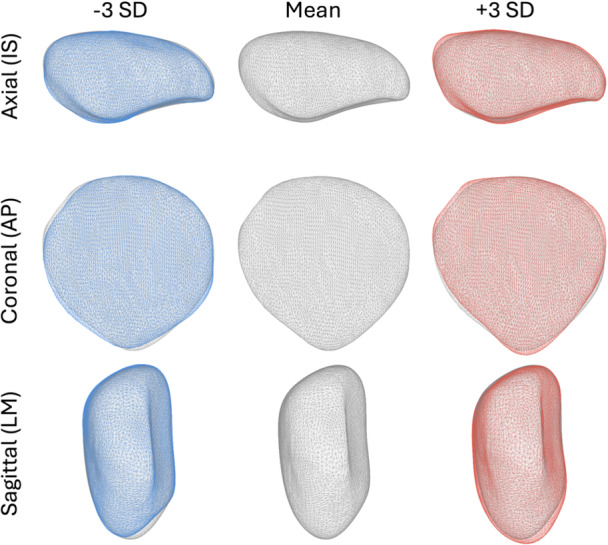
Patella mode 3 surface deformation rendered at −3 SD (blue, left), the population means (gray, center), and +3 SD (red, right). Rows show axial (IS, top), coronal (AP, middle), and sagittal (LM, bottom) views. Each deformed surface is drawn on top of a faint wireframe of the population mean shape, so the displacement is visible against a shared reference. Patella mode 3 primarily captured asymmetric patellar facet and ridge morphology. The −3 SD direction, which was observed in the recurrent dislocation group, corresponded to a more asymmetric patellar shape with a shifted ridge and altered facet curvature.

Patella mode 3 was a statistically significant mode that separated the recurrent dislocation group from the control and single‐dislocation groups, as shown in Figure 6b. The recurrent dislocation group had significantly smaller mode 3 coefficients compared to the other groups. Figure 8 shows the patella mode 3 deformation as −3 SD, mean, and +3 SD surface meshes with the mean shape overlaid. The −3 SD direction, which corresponds to the lower mode 3 scores observed in the recurrent dislocation group, primarily represents asymmetric patellar facet and ridge morphology, including a shifted and more prominent patellar ridge and altered medial‐lateral facet curvature. Therefore, compared to the control and single dislocation groups, the recurrent dislocation group showed a more asymmetric patellar shape rather than a simple global size difference.

## 4 Discussion

This study has illustrated the significance of MRI‐based 3D Statistical Shape Modeling (SSM) in differentiating between patellar dislocation patients and control subjects, as well as among patients with single versus recurrent patellar dislocations. The findings underscore the role of specific bone shape features in the pathology of patellar dislocations and their potential to guide clinical decision‐making.

The observed differences in femur and patella bone shapes among the groups highlight the potential biomechanical and anatomical factors contributing to patellar dislocations. The fact that certain modes were significantly different between groups indicates that these shape changes could serve as markers for identifying individuals at higher risk of initial or recurrent dislocations. For instance, the significant change in mode 4 for the femur among dislocation groups suggests a known relationship between the trochlea groove depth and patellar dislocation risk. More interestingly, the variations in patella shape, particularly the concavity of the lateral facet and the shape of the patellar ridge, which may be related to patellar tilt and the medial facet being unloaded, point to the importance of the patella's anatomy in dislocation episodes. These bone shape features could have implications for surgical planning and post‐dislocation management, aiming to restore normal joint anatomy and function as much as possible to prevent further dislocations.

Some of these features have been observed in patients with instability and other pathologies. For instance, a shallow trochlea groove, known as trochlear dysplasia, is strongly associated with patella instability [35], where the authors reported that 96% of the patients with confirmed patellar dislocation had trochlear dysplasia. It was also reported to be associated with higher Whole‐Organ‐MR‐Imaging Score and smaller cartilage volume, indicating more severe osteoarthritis within the patellofemoral joint [36]. Similarly, the shifted patellar ridge and hook shaped patella lateral facet were identified as significant predictors of knee OA progression at the patellofemoral joint over 3 years [29].

This investigation into the bone shape changes associated with single and recurrent patellar dislocations, using MRI‐based 3D SSM, has provided valuable insights into the anatomical differences characterizing these conditions. The study has identified specific bone shape features that distinguish between control subjects and dislocation patients, as well as between single and recurrent dislocation cases. The identification of these key bone shape features associated with dislocation risk holds promise for improving patient outcomes through the early identification of individuals at risk and the implementation of personalized treatment plans.

Our results are broadly consistent with the recent MRI‐based statistical shape analysis by Nagawa et al., who compared femurs with patellofemoral instability with normal femurs and reported that 3D femoral shape information could distinguish patellofemoral instability (PFI) from normal anatomy [37]. The present study extends this line of work in two ways. First, we evaluated both femoral and patellar shape rather than femoral shape alone. Second, we separately examined single‐ and recurrent‐dislocation groups, allowing us to identify candidate shape features associated not only with patellar dislocation status but also with recurrence. The recurrent‐dislocation features identified here, including altered lateral femoral condyle geometry and patellar lateral‐facet/ridge morphology, may therefore provide complementary information to prior PFI‐versus‐control shape analyses.

One of the limitations of this study is the relatively small patient cohort. Future research should focus on expanding the application of SSM to larger, more diverse cohorts to validate these findings and explore the potential for integrating bone shape analysis into routine clinical practice. Another limitation is that single dislocators could go on multiple dislocations beyond the study period, redefining them into the recurrent dislocation group. Therefore, longitudinal studies with longer study period examining the evolution of bone shape changes post‐dislocation and post‐treatment could provide further insights into the natural history of patellar dislocations and the effectiveness of different management strategies. Furthermore, this study considered femur and patella bone SSM features independently. Applying SSM to femur and patella together may provide additional bone shape features about the interaction between femur and patella that could better distinguish between the control and dislocation groups, as well as the single and recurrent dislocation groups. Finally, because this exploratory study tested multiple retained principal‐component modes without multiple‐comparison correction, the statistically significant modes should be interpreted as candidate shape features that require validation in larger independent cohorts.

## 5 Conclusion

With the 3D SSM modeling for femur and patella, we identified visible anatomical differences among all 3 populations: control, single, and recurrent patellar dislocation. Some bone shape features showed significant differences between the dislocation and control groups. Moreover, the current SSM also uniquely identified anatomical features distinguishing between patients’ knees with single and recurrent dislocations. Improved understanding of these bone shape features can help to better identify patients at risk of patella dislocation, as well as those most likely to experience recurrent dislocations following conservative treatment. This should help clinicians stratify patients, optimize their treatment and clinical management plans, and thus minimize functional impairment and patellofemoral cartilage damage.

## Author Contributions

Substantial contributions to research design, or the acquisition, analysis or interpretation of data: Mingrui Yang, John J. Elias, Mei Li, Jin Ma, Ceylan Colak, Carl S. Winalski, LDF, Xiaojuan Li; drafting the paper or revising it critically: Mingrui Yang, John J. Elias, Xiaojuan Li; approval of the submitted and final versions: all authors.

## Data Availability

The data that support the findings of this study are available on request from the corresponding author. The data are not publicly available due to privacy or ethical restrictions.
